# Reactive oxygen species and its role in pathogenesis and resistance to therapy in acute myeloid leukemia

**DOI:** 10.20517/cdr.2023.125

**Published:** 2024-02-22

**Authors:** Jamshid Sorouri Khorashad, Sian Rizzo, Alex Tonks

**Affiliations:** ^1^Department of Immunology and inflammation, Imperial College London, London, W12 0NN, UK.; ^2^Department of Molecular Pathology, Institute of Cancer Research, Sutton, SM2 5PT, UK.; ^3^Department of Haematology, Division of Cancer & Genetics, School of Medicine, Cardiff University, Cardiff, CF14 4XN, UK.

**Keywords:** Reactive oxygen species, acute myeloid leukemia, NOX2, drug resistance, relapse

## Abstract

Relapse following a short clinical response to therapy is the major challenge for the management of acute myeloid leukemia (AML) patients. Leukemic stem cells (LSC), as the source of relapse, have been investigated for their metabolic preferences and their alterations at the time of relapse. As LSC rely on oxidative phosphorylation (OXPHOS) for energy requirement, reactive oxygen species (ROS), as by-products of OXPHOS, have been investigated for their role in the effectiveness of the standard AML therapy. Increased levels of non-mitochondrial ROS, generated by nicotinamide adenine dinucleotide phosphate oxidase, in a subgroup of AML patients add to the complexity of studying ROS. Although there are various studies presenting the contribution of ROS to AML pathogenesis, resistance, and its inhibition or activation as a target, a model that can clearly explain its role in AML has not been conceptualized. This is due to the heterogeneity of AML, the dynamics of ROS production, which is influenced by factors such as the type of treatment, cell differentiation state, mitochondrial activity, and also the heterogeneous generation of non-mitochondrial ROS and limited available data on their interaction with the microenvironment. This review summarizes these challenges and the recent progress in this field.

## INTRODUCTION

Acute myeloid leukemia (AML) is the most common acute leukemia and constitutes a significant portion of all leukemia with a poor outcome. Resistance to therapy, either primarily or following a remission period, is the main challenge for the management of AML patients and, indeed, all cancers. Therefore, understanding the mechanism of emerging resistance and relapse is one of the main research topics, especially for cancers with high rates of relapse and mortality, such as AML. AML is characterized by the acquisition of gene mutations or chromosomal abnormalities that induce proliferation and disturb differentiation of hematopoietic progenitor cells (HPC), leading to the clonal expansion of myeloid stem and progenitor cells in the bone marrow (BM)^[1]^. AML standard induction therapy is based on using cytosine arabinoside (ARA-C) and anthracyclines such as daunorubicin (DNR) or idarubicin. This treatment has effectively remained unchanged for decades, although within recent years, it has been combined with additional targeted therapies depending on the clinical condition and leukemia genotype. Some of these therapies have been approved by the FDA, such as inhibitors targeting FMS-like tyrosine kinase 3 (FLT3), B-cell lymphoma 2 (BCL-2) and isocitrate dehydrogenase 1/2 (IDH1 and IDH2) while many others are currently under investigation through clinical trials such as the inhibitors of TP53, NEDD8-activating enzyme (NAE) and cyclin-dependent kinase (CDK)^[2]^.

Standard induction therapy leads to complete remission, determined clinically, morphologically, immunologically, and sometimes at the molecular level, in 40% to > 90% of cases, depending on patient age and the presence or absence of specific somatically acquired genetic alterations. However, at least 50% of younger patients and 80% of those older than 60 years will experience relapse within 5 years^[3-7]^. Together with post-remission therapy such as additional chemotherapy and/or hematopoietic stem cell (HSC) transplantation, 5-year survival rates of < 5%-20% and > 40% are achieved for patients older and younger than 60 years, respectively^[8-10]^. In most cases, it has been shown that the founder leukemic clone persisted following chemotherapy and established the basis of relapse years later^[11]^. Understanding the operating mechanisms that protect AML cells against induction therapy is essential for designing new therapies to eradicate these cells in order to prevent relapse. Targeting leukemia stem cells (LSC) as the source of relapse is a current topic of mainstream AML research. LSC refer to a subpopulation of leukemia cells that have a self-renewal capacity, fail to properly differentiate into mature hematopoietic cells, exhibit quiescence and survival signaling to support their viability, and are not eradicated by conventional chemotherapy^[12-14]^. LSC may develop from HSC or HPC that acquire self-renewal capacity following genomic aberrations^[15-18]^. Because of the similarities to normal HSC, the specific cell surface markers have not been completely defined for LSC. However, exploration of AML patients’ blasts has led to the identification of a variety of markers, which are used for characterization of the cell populations enriched in LSC and are updated as new discoveries emerge^[19-21]^. Various mechanisms have been proposed for drug resistance in LSC, including quiescence to avoid targeting cell cycle^[22]^, high self-renewal capacity to allow repopulation of leukemia cells following therapy^[23,24]^, expressing high levels of transporters to pump drugs out of cells^[25,26]^, altering drug metabolism to inactivate chemotherapy agents^[27,28]^, activation of survival signaling pathways such as the signals mediated by WNT/β-catenin, Hedgehog, BCL-2^[29-31]^, and interacting with stromal cells in the leukemic niche to receive signals that mediate adhesion-related drug resistance^[32,33]^.

One of the proposed metabolic pathways to be involved in resistance to therapy is the alteration in redox metabolism and, in particular, oxidation. Malignancies including AML have been observed to be in a state of redox imbalance where the homeostasis between oxidants and antioxidants has shifted, often to the extent that cancer cells have increased oxidants within the cell. Oxidative phosphorylation (OXPHOS) is the process by which nutrients such as carbohydrates, lipids, and amino acids are broken down into usable energy within the mitochondria. OXPHOS is the target for Venetoclax (VEN), a potent and selective inhibitor of BCL-2, which was approved in 2018 by the FDA in combination with either DNA methyltransferase inhibitors or low-dose ARA-C for the management of older or unfit AML patients^[34,35]^. VEN can impair oxidative phosphorylation in AML cells through mitochondrial effects and lowering amino acid uptake^[36]^, which adds to its antileukemic activity beyond just BCL-2 inhibition. Despite promising responses in various studies, resistance to VEN-based combinations can emerge in AML patients. In phase 1/2 trials of VEN and hypomethylating agents, the overall response (complete remission with or without complete hematology recovery) rate was around 60%-70% in treatment-naïve elderly AML patients. However, 30%-40% of patients exhibited primary resistance and did not respond to the combination therapy^[37,38]^. The majority of AML patients (70%-90%) who initially responded to VEN combination therapy relapsed within approximately 1 year of beginning treatment, indicating acquired resistance develops in many patients^[38,39]^. As a target of VEN, alterations to OXPHOS and its regulation in AML cells are expected to play a role in contribution to resistance. To understand the role of oxidation in the development of resistance to standard induction therapy or combined VEN in AML and other new inhibitors, an understanding of the role of oxidation in cell signaling, normal hematopoiesis, and the role played by different sources of reactive oxygen species (ROS), the mediator of oxidation, is required.

## ROS GENERATION AND HOMEOSTASIS

ROS refers to various oxygen-containing free radical species and other reactive molecules that are more reactive than dioxygen (O_2_). The main species include superoxide anion (O_2_^•-^), hydrogen peroxide (H_2_O_2_), hydroxyl radicals (OH^•^), and singlet oxygen (^1^O_2_). H_2_O_2_ is the most important intracellular ROS, which has the capacity to cross the biological membrane by passive diffusion or facilitated transport via channels, such as aquaporins, and reversibly oxidize the cysteine residues in proteins^[40]^. H_2_O_2_ exhibits lower overall reactivity in comparison to O_2_^•-^ and has high selectivity for the thiol group of cysteine residues and displays the greatest stability compared with other physiologically relevant ROS^[41]^.

Physiologically, ROS are initially generated via the univalent reduction of O_2_, which generates O_2_^•-^. Superoxide subsequently dismutates to H_2_O_2_ through the catalytic activity of superoxide dismutase (SOD) enzymes^[42]^. The main cellular sources of ROS generation include mitochondria, where O_2_^•-^ is generated through the reaction of O_2_ with electron at complex I/III at electron transfer chain, endoplasmic reticulum, and from nicotinamide adenine dinucleotide phosphate (NADPH) oxidase (also known as NOX)^[42,43]^. The NOX family consists of family members NOX 1, 2, 3, 4 and 5 and dual oxidases (DUOX) 1 and 2. H_2_O_2_ is mainly produced by NOX4, DUOX1, DUOX2, and SOD2^[44]^. The structural differences among the members of the NOX family correlate to the specific functions. The presence of a calcium binding region in the N-terminus of DUOX1, DUOX2, and NOX5 is a distinguishing feature of these proteins from the rest of the NOX family, and the presence of a domain with similarity to the active site of peroxidase in DUOX1 and DUOX2 distinguishes these two from NOX5^[45]^. Regarding the type of ROS generation, NOX1, NOX2, NOX3 and NOX5 generate O_2_^•-^ while NOX4, DUOX1 and DUOX2 produce H_2_O_2_^[46]^. Each member of the NOX family is a protein complex composed of several components, for example, p22phox, gp91phox protein (membrane proteins), p47phox, p67phox, p40phox (cytosolic proteins), and the GTP-binding protein Rac1/2 forming the NOX2 complex. Once activated, cytoplasmic protein subunits translocate to the membrane, undergo a conformational change, and form a complex with membrane subunits to activate the enzyme complex. The components of the NOX complex influence the subcellular distribution and the mechanism of regulation^[45]^. The other minor sources of ROS generation that do not contribute significantly to the pool of the ROS in cells are from the activities of the following enzymes: cytochrome P450 (CYP), aldehyde oxidase, glycolate oxidase, monoamine oxidase, xanthine oxidase, hydroxyacid oxidase, cyclo- oxygenase (COX), and amino acid oxidase^[47]^.

The main sources of ROS production in normal hematopoietic cells under physiological conditions are (i) through conversion of 0.1%-0.2% of the consumed O_2_ to ROS in mitochondria; and (ii) through the activity of plasma cell membrane-bound protein NOX family (extramitochondrial)^[48]^. Members of the NOX family are found on CD34^+^ hematopoietic cell membranes, with NOX2 as the most abundantly expressed family member^[49]^.

## PROTEIN MODIFICATION BY ROS

In the previous two decades, it has become clear that in addition to its canonical role in cellular defense against infection, ROS also play a significant role in cell signaling. It is important to note that extracellular H_2_O_2_ is readily transported across the cell membrane via the transmembrane water permeable channel protein family of aquaporins^[41]^. Either intracellular or extracellular ROS has the capacity to induce post-translational modifications (PTM). These have been observed in proteins involved in all aspects of cellular processes and could be exacerbated under oxidative stress when an imbalance between ROS generation and antioxidant defense systems leads to the accumulation of ROS. The ROS-induced PTM can be either reversible or irreversible and highly regulated under normal physiological conditions. The modifications of the proteins by ROS influence key functions such as phosphorylation, acetylation, ubiquitination, *etc.*^[50]^. One of the two main ways that ROS alter the function of the involved proteins in cell signaling is by oxidation of the thiol functional group of the cysteine residues^[51,52]^. The pattern of cysteine residues as an active site or co-factor binding site in proteins is highly conserved, explaining its significance as a target for ROS as a main player in various cellular processes^[53]^. The other way ROS alter protein function is to affect intra- and inter-cysteine disulfide bond formation, which, depending on the affected protein, may lead to its increased or decreased activity^[50]^. If the cysteine is involved in the formation of the active site in a protein, ROS-induced disulfide bond formation may switch the activity on or off; alternatively, disulfide bond formation may change the activity of a protein by changing its structure^[54]^. Among the mechanisms, disulfide bond formation influences signaling pathways and cell cycle and their role in the shuttling of the transcription factor from the cytoplasm to the nucleus in response to elevated H_2_O_2_ concentrations^[55]^ and remodeling of the cytoskeleton during phagocytosis^[56]^. In addition to protein modifications, ROS can have a direct impact on the structure of DNA. Reactions of ROS with DNA can generate numerous oxidized bases, including 8-hydroxy-2-deoxyguanosine (8-OHdG) which causes G:C to T:A DNA transversions and particularly has been observed in association with relapse in AML and has an inverse relationship with the total antioxidant capacity of the cell^[57]^. Table 1 provides examples of cysteine modifications by ROS and the resulting effects on proteins, organized by protein functional category.

**Table 1:** Cysteine modification by ROS and the resulting effect on protein function

**Protein/Pathway**	**Effect**
PTP	Oxidation of the catalytic cysteine inactivates PTPs, allowing sustained tyrosine phosphorylation and cell signaling. PTEN^[58]^, PTP1B^[59]^, and SHP-2^[60]^ are among the known PTPs whose activities are inhibited through this mechanism
Transcription factors	Cysteine oxidation can inhibit DNA binding activity. One of the well-known proteins for having altered function due to this mechanism is TP53^[61]^
Ion channels and receptors	Cysteine oxidation has been shown to modulate the activity of some protein channels such as Ryanodine receptor^[62]^ and NMDA receptor^[63]^
Metabolic enzymes	Oxidation provides redox control over certain metabolic fluxes. For example, cysteine residues of certain PRX undergo reversible oxidation to sulfinic acid (Cys-SO2H) which contributes to regulation of its function^[64]^ or oxidation of the cysteine residue (Cys358) by H_2_O_2_ inhibits the activity of PKM2, an enzyme that catalyzes the final rate-limiting reaction in glycolysis^[65]^
Protein kinases	Oxidation may regulate the activity of some kinases. For example, it has been shown to increase the activity of ASK1^[66,67]^
Regulator proteins	Oxidation may control the function of some proteins by inhibiting their interactions with other molecules; for example, oxidation of cysteine residue (Cys374) in actin prevents the interaction of G-actin subunits and this disrupts filament formation^[68]^
Signaling proteins	Oxidation can modify the structure of a protein by forming intermolecular disulfide bonds between conserved cysteine residues within a protein, leading to the change of function and subsequently downregulation or upregulation of the downstream signaling pathways. For example, JNK is a family of stress-activated protein kinases that play important roles in regulating cell proliferation, differentiation, apoptosis, and other cellular processes. High ROS induces intermolecular disulfide bonds between conserved cysteine residues in the activation loop of JNK. This prevents the access of phosphatase to activation loop, leading to sustained JNK activation^[69]^

ROS: Reactive oxygen species; PTP: protein tyrosine phosphatases; PTEN: phosphatase and tensin homolog; PTP1B: protein tyrosine phosphatase 1B; SHP-2: SRC homology 2 (SH2), refers to protein Tyrosine-protein phosphatase non-receptor type 11, also known as PTPN11; NMDA: N-methyl-D-aspartate; PRX: peroxiredoxins; H_2_O_2_: hydrogen peroxide; PKM2: pyruvate kinase, isoform 2; ASK1: apoptosis signal-regulating kinase 1; JNK: c-Jun N-terminal kinase.

## THE ROLE OF ROS IN NORMAL HEMATOPOIESIS

Cell differentiation and proliferation are intimately linked and associated with enhanced metabolic activity and higher ROS levels. In contrast, HSCs have a low-oxidative metabolism leading to low production of ROS and clearly have a less phenotypic differentiation state^[49]^. The association of HSC with lower levels of ROS is linked with their location in the BM as the non-dividing HSC reside in the hypoxic microenvironment of the BM which contributes to their maintenance, and their metabolism which is based on anaerobic glycolysis rather than OXPHOS. The hypoxic microenvironment of the BM includes a large proportion of the extravascular compartment, and in particular, peri-sinusoidal region is the most hypoxic region^[49]^. In addition to the peri-sinusoidal region, most of the endosteal niches are also hypoxic due to being relatively far from blood vessels^[70]^. Hypoxia through activation of hypoxia-inducible factor (HIF)-1α induces a quiescence phenotype and metabolism switching to glycolysis in HSC^[71]^. The level of ROS remains low in HSC and common myeloid progenitors (CMP) but increases during differentiation to granulocyte-monocyte progenitors (GMP). In contrast, intracellular levels of ROS remain as low as HSC in megakaryocyte-erythrocyte progenitor cells (MEP)^[72]^. The regulation of ROS levels is essential for the maintenance of HSC as increased levels of ROS induce cell cycling and differentiation through activation of p38^MAPK^ and mTOR pathway, which has a negative impact on self-renewal of HSC, and if it is not controlled, it leads to premature exhaustion of HSC^[71]^. To keep the ROS levels under control, aerobic organisms employ the following defense mechanisms: (i) glutathione peroxide; (ii) peroxiredoxin; (iii) catalase; and (iv) thioredoxins^[73]^. HSCs protect themselves against ROS mainly through FOXO transcription factors, in particular FOXO3, which activates antioxidant molecules such as dismutase and catalase^[49]^. By provoking base excision repair of oxidative DNA damage and regulation of mitochondrial oxidative metabolism, FOXO3 protects HSC^[74]^. Through a different pathway, FOXO3 acts as a major regulator of autophagy and mitophagy, and this protects hematopoietic cells as these processes remove damaged mitochondria and toxic proteins^[75]^. In addition, FOXO1, FOXO3, FOXO4, and FOXO6 induce transcription of genes that eliminate ROS such as *SOD2*, *CAT*, *PRDX3*, and *SENP*^[76]^. Furthermore, they decrease the level of free transition metal ions by elevating metallothionein and ceruloplasmin^[77]^ and also have a role in the regulation of the genes involved in cell-cycle arrest and apoptosis^[76]^.

The NOX family is important in normal hematopoiesis as it has been shown that the deficiency in some members of this family, such as NOX2 and GTPase RAC proteins, disturbs the balance between self-renewal and differentiation of hematopoietic cells and leads to damaged hematopoiesis^[78-81]^. The study by Hole *et al.* demonstrated the induction of excessive ROS, generated by NOX2 in normal HSC, promoted growth factor-independent proliferation^[82]^, and the report from Adane *et al*. showed the deficiency of NOX2 in HSCs led to loss of their self-renewal capacity, and impaired differentiation with a tendency towards myeloid lineage^[78]^. As the current methodologies are not entirely reliable for distinguishing NOX2-derived ROS from mitochondrial-derived ROS, the contribution from each of these sources (NOX or mitochondria) to ROS generation in HSC and their significance in the maintenance of stemness, proliferation and differentiation remains to be discovered. The mechanisms through which ROS contribute to stemness maintenance in HSC and also proliferation and differentiation remain to be understood. It can be hypothesized that there is a ROS concentration-dependent mechanism where low ROS levels alter residues in proteins (resulting in higher activity) that are involved in stemness, but when ROS levels increase, residues in proteins involved in proliferation are modified with subsequent functional activation^[42]^. FOXO transcription proteins are examples of the proteins in HSC that can be activated by ROS, and their activity contributes to stemness^[83]^ while ROS-mediated alterations to a protein such as PTPMT1 may block HSC differentiating^[84]^. The investigations, focusing on measuring ROS level fluctuations and correlating them with the modified functions of various proteins in HSC, would provide evidence to verify this hypothesis. This hypothesis is further supported by a recent leukemia model-based study that AML cells with low ROS were enriched within the leukemia-initiating cells that had higher percentages of CD34^+^CD38^-^ cells, reflecting the reverse association between ROS level and differentiation^[85]^.

## STRESS RESPONSE TO ROS IN HEMATOPOIETIC CELLS

**Figure 1:**
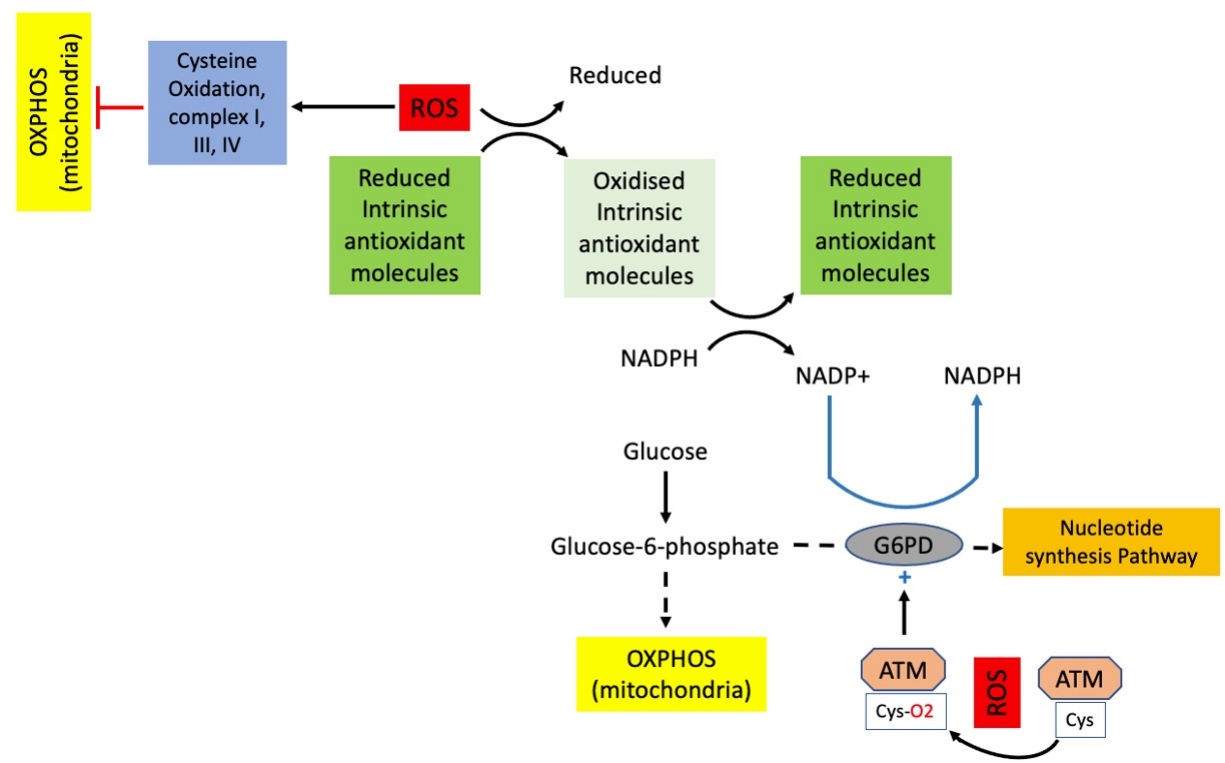
Short-term response to oxidative stress. The initial response to oxidative stress primarily relies on the activity of antioxidant molecules, which donate electrons to ROS to reduce the oxidative state. To continue functioning as antioxidants, these molecules must be replenished, by receiving electrons from NADPH. ROS, through the oxidation of cysteine residues on the ATM protein, leads to increased ATM activity. In turn, ATM activates the G6PD enzyme, which enhances the production of NADPH from NADP^+^. This process has two immediate consequences: the generation of more NADPH and the redirection of glucose-6-phosphate from oxidative phosphorylation in the mitochondria to the nucleotide synthesis pathway. Consequently, this results in reduced ROS production. Additionally, ROS has a direct impact on mitochondria by oxidizing cysteine residues in complexes I, III, and IV, leading to decreased oxidative phosphorylation. ROS: Reactive oxygen species; NADPH: nicotinamide adenine dinucleotide phosphate; ATM: Ataxia Telangiectasia Mutated; G6PD: glucose-6-phosphate dehydrogenase; NADP^+^: nicotinamide adenine dinucleotide phosphate.

The regulatory antioxidant mechanisms that keep ROS levels under control depend on the cell type and amount of ROS required for the activity of certain signaling pathways. If the produced ROS exceeds the capacity of antioxidant systems in the cell, oxidative stress occurs^[71]^. In cancer cells, a high proliferative state requires enhanced metabolism, which in return requires higher production of ATP. The increased production of ATP through OXPHOS in the mitochondria is accompanied by increased levels of ROS as a result of this process. ROS as mediators in certain signaling pathways have pro-tumor activity and their elevation contributes to the pro-survival and proliferation of cancer cells. As ROS are toxic to cells beyond a certain threshold, the cancer cells increase their antioxidant capacity to combat the deleterious effects of excessive ROS, which include DNA damage. ROS may act as oncogenic or tumor-suppressive molecules depending on the balance between ROS and antioxidants. In some cancer cells, by activating the signaling pathways that regulate proliferation and survival, ROS contribute to the initiation and progression of cancer and, therefore, are considered oncogenic^[86-88]^. However, ROS induce cell cycle arrest or apoptosis when exceeding a certain level and therefore can be considered tumor-suppressive^[89,90]^. ROS accumulation can damage cells and lead to cell death through several pathways, such as damaging DNA and subsequent G2/M phase arrest through the activation of ATM^[91]^, MAPK-mediated induction of mitochondrial-dependent apoptosis, and activation of the proapoptotic proteins of the BCL2 family leading to mitochondrial membrane permeabilization and cell death^[92-95]^. To maintain the ROS levels within oncogenic activity range and avoid death, the cancer cells upregulate antioxidant pathways^[96]^. If the ROS levels exceed the antioxidant capacity, then a stress response to survive is initiated and this includes a short-term metabolic [Figure 1] or a long-term genetic reprograming^[97]^ [Table 2].

**Table 2:** Transcription factors that are activated as a genetic reprograming adaptive response to chronic exposure to ROS

**Gene**	
*NRF2*	First-tier defense (The principle inducible defense against oxidative stress) Downregulation of ROS production by suppressing the expression of NOX4, IL-1B, IL-6, *etc*. Upregulation of serine synthesis which leads to the production of GSH
*AP-1*	Second-tier defense Induction of the genes that: (1) scavenge ROS; (2) synthesize GSH; and (3) suppress the level of free iron
*NF-κB*	Second-tier defense It not only regulates the expression of antioxidant genes but also the expression of pro-oxidant genes such as *CYP2E1*, *NOX2*, *XOR*, *NOS2*, *COX2*, *ALOX5*, and *ALOX12*
*FOXO*	Induction of the genes that (1) eliminate ROS; (2) improve the mitochondrial Redox; and (3) suppress free transition of metal ions
*HIF1-α*	Regulate the expression of the antioxidant genes under hypoxia By inducing the genes encoding for lactate dehydrogenase and pyruvate dehydrogenase kinase, the reactions shift from TCA in mitochondria to lactate production and, as a result, reduction of ROS production by mitochondria
*PGC-1α*	It can increase antioxidant capacity and decrease the production of ROS by mitochondria through mitochondrial biogenesis and also activate uncoupling proteins
*HSF1*	Induction of antioxidant gene and also induction of heat shock protein
*TP53*	Through regulation of various genes with a wide range of activity from scavenging ROS, supporting GSH, to the third-tier defense which is apoptosis Under mild oxidative stress: TP53 induces gene expression leading to adaptatio Under high oxidative stress: TP53 activates the pathways leading to apoptosis

ROS: Reactive oxygen species; GSH: glutathione; HIF: hypoxia-inducible factor; TCA: tricarboxylic acid cycle.

The most abundant antioxidant molecule in the cell is glutathione (GSH), which is used by GSH-S transferases and GSH peroxidases to reduce ROS. There are two other significant antioxidant networks, sulfiredoxin (Srx) and thioredoxin (Trx), which have a role in the clearance of ROS, but they are less abundant^[98,99]^. During oxidative stress, GSH and Trx serve as the major defensive molecules to protect cells. For these molecules to function effectively, they need to be in their reduced state, which is facilitated by NADPH (nicotinamide adenine dinucleotide phosphate). In times of oxidative stress, when NADPH is consumed to maintain GSH and Trx in their reduced state, the production of NADPH is stimulated by activating glucose-6-phosphate dehydrogenase (G6PD). This is a key enzyme in the pentose phosphate pathway (PPP). The inhibitory effect of NADPH on G6PD is removed when its level is reduced, and glucose metabolism shifts from glycolysis through the oxidative arm of the PPP towards nucleotide synthesis, thereby generating NADPH^[97]^. It has been shown that ROS can modify various cysteine residues within protein subunits of the mitochondrial respiratory chain complex. This modification has been observed to result in a reduction of OXPHOS, and it has been suggested as one of the mechanisms involved in the cellular stress response^[97,100,101]^. When the levels of ROS remain elevated for an extended period, the cell responds by modifying the transcription of genes involved in controlling intracellular redox balance. Key regulatory genes in this process include *NRF2* (the first-tier defense), *AP-1*, *NF-κB* (the second-tier defense), *HSF1*, *HIF-1α*, and *TP53*^[97,102]^.

## THE ACTIVITY OF ROS IN AML

In contrast to normal hematopoiesis, OXPHOS is the main source of energy production in AML cells^[103]^. LSC and their progeny have a greater mitochondrial mass and a higher rate of O_2_ consumption compared to normal HPC^[104]^. Compared to normal HSC, AML cells produce higher levels of ROS^[105]^. CD34^+^ AML blasts were shown to be categorized as high and low-level ROS^[106]^ (similar to normal marrow) with different characteristics in regard to gene expression, absolute mitochondrial content (reduced in low-ROS), ATP content (reduced in Low-ROS), cell size (reduced in Low-ROS), sensitivity to VEN (enhanced in Low-ROS), and percentage of CD38^-^ marker (high in Low-ROS)^[107]^. LSC in AML patients are mainly enriched in low-ROS CD34^+^ cells^[106,108]^. Transcriptome analysis of Low-ROS CD34^+^ cells in AML patients demonstrated gene expression patterns associated with increased stemness and reduced differentiation and apoptosis pathways compared to high-ROS CD34^+^ cells^[107]^.

The increased levels of ROS (mainly O_2_^•-^) in AML patients have been shown to be a product of NOX family activity rather than mitochondria^[49]^. Increased NOX2 activity was reported in 60% of AML cells from patients, attributed to enhanced ROS. In AML cells, NOX2-induced ROS promote proliferation^[105]^ and also alter transcription of the genes involved in carbohydrate metabolism due to enhanced glucose uptake^[109]^. According to the latter study, NOX2-induced ROS increased the intermediate metabolites required for the glycolysis and enhanced glucose uptake by upregulating the expression of 6-phosphofructo-2-kinase/fructose-2,6-bisphosphatase (PFKFB3).

**Figure 2:**
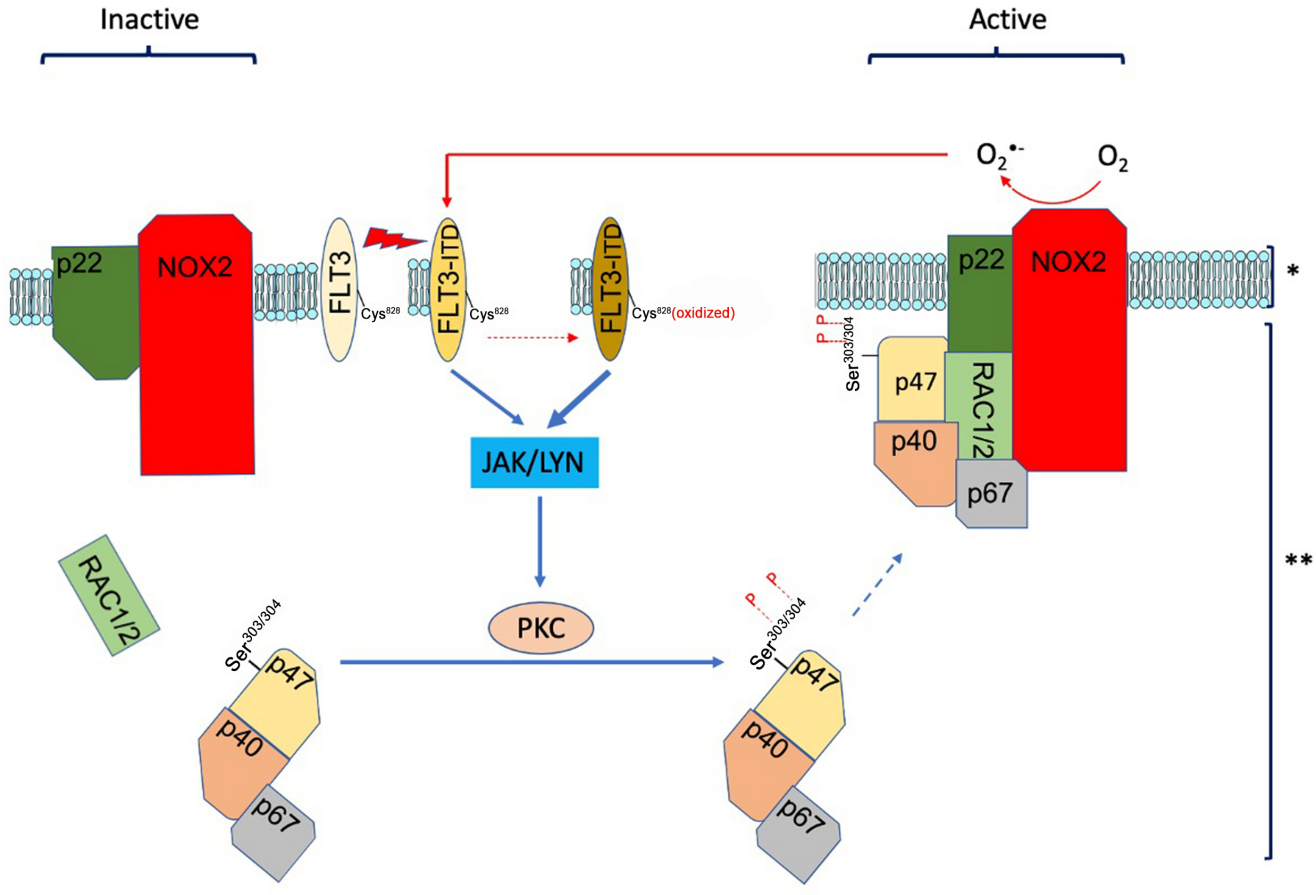
Mutant FLT3 and its role in ROS generation through the NOX2 complex. The membrane (p22phox and NOX2) and cytoplasm (p47phox, p67phox, p40phox) components and the GTP-binding protein Rac1/2 form the NOX2 complex upon activation. FLT3-ITD phosphorylates JAK/LYN, which subsequently phosphorylates and activates PKC. The activated PKC phosphorylates serine residues 303 and 304 on p47, leading to its activation. As a regulator of the NOX2 complex, the phosphorylated form of p47 activates NOX2, resulting in the generation of superoxide and subsequent ROS via NOX2 activity. The generated ROS oxidizes cysteine 828 on FLT3-ITD (and possibly other serine residues). Cysteine 828 has been shown to play a significant role in enhancing the transforming activity of FLT3, and its oxidation is expected to further increase FLT3 activity. This series of events creates a feedback loop, resulting in elevated FLT3 activity and increased ROS production as outcomes. *: plasma membrane; **: cytoplasm; O_2_^•-^: superoxide anion; P: phosphorylation. FLT3: FMS-like tyrosine kinase 3; ROS: reactive oxygen species; GTP: guanosine triphosphate; ITD: internal tandem duplication; JAK: janus kinase; LYN: LYN proto-oncogene; PKC: protein kinase C.

Among the NOX family, NOX2 expression mainly correlates with superoxide generation in primary AML blasts. RAS or FLT3-ITD mutations have been associated with high levels of ROS in AML, although this association is not specific to these mutant genes and higher levels of ROS have been correlated with other oncogene driver mutations in myeloid malignancies^[105,110]^. NOX-generated ROS appear to be a primary source of ROS in FLT3-ITD-expressing AML cells as these cells produce increased levels of NOX2 and NOX4 and their partner protein p22^phox^ compared to wild-type FLT3 cells^[111]^. The enhanced ROS formation in FLT3-ITD-expressing AML cells promotes proliferation and migration and thereby contributes to leukemic cell transformation^[112,113]^. The recent investigation of the mechanism through which FLT3*-*ITD increases ROS by Germon *et al.* suggests that FLT3-ITD activity modifies and activates the regulatory units of NOX2, leading to enhanced activity of NOX2 and consequently increases ROS levels. The increased ROS, in turn, oxidizes certain residues on FLT3 (Cys828 and Tyr842) and other kinases, leading to enhanced feedback^[114]^ [Figure 2]. A positive correlation between the increasing levels of ROS within the mitochondria and enhanced sensitivity of AML cells to FLT3 inhibitors is in line with this defective positive regulation loop connecting FLT3 activity and ROS production^[115]^. Animal studies by Aydin *et al.* indicated that the generated ROS in *KRAS* mutant leukemia cells derive from the activity of NOX2 and contribute to the progression of KRAS-induced leukemia^[116]^. The key role of NOX2 in energy metabolism was investigated further in a recent study by Ijurko *et al.*, where the removal of NOX2 in an *in vitro* AML model led to reduced glycolysis and mitochondrial respiration, while enhancing fatty acid metabolism as a source of energy production indicating the generated ROS by NOX2 has a regulatory influence on the metabolic pathway in mitochondria^[117]^.

## RESISTANCE TO THERAPY AND THE ROLE OF ROS

Remission in AML patients refers to the reduction or absence of signs and symptoms of the disease following treatment and can be either a complete remission (CR) or partial remission (PR). CR is defined by a reduction of BM blasts to less than 5%, no blasts in peripheral blood (PB), full recovery of PB cell counts, and no signs or symptoms of disease, while in PR, blasts are decreased by more than 50% in BM compared to pre-treatment counts, also reduced in PB and there is some improvement in blood counts. Resistance refers to when leukemia cells do not respond to anti-cancer treatments and can be categorized into primary and secondary. Primary resistance (or refractory AML) is defined by the failure of AML blast cells to respond to initial treatment and, under the ELN criteria, refers to patients who fail to meet the CR criteria after receiving the standard 2 cycles of anthracycline/ARA-C double induction chemotherapy^[118]^. Secondary or acquired resistance develops when a patient who previously responded to treatment relapses. In secondary resistance, leukemia cells become resistant to therapies that initially worked. The ELN defines secondary/acquired resistance in AML as leukemic relapse after having initially responded to treatment and achieved CR, with relapse timeframes varying based on cytogenetic risk profile^[118]^. There is no universally accepted standard definition for drug/therapy resistance in AML mouse models. However, drug resistance in AML mouse models is generally determined by failure to achieve a reduction in BM blast counts and no improvement in survival compared to untreated mice. The initial response to treatment followed by eventual progression or repopulation of leukemia cells is considered relapse in mouse models. Specific quantitative criteria depend on the applied model system and the treatments.

LSC are suspected to be the main cell population which are not affected by chemotherapy, leading to relapse months or years later^[119]^. The contribution of ROS to drug resistance is thought to be influenced by its concentration, the underlying source of production, or the type of treatment, and therefore, it is complex.

### ROS level

Failure to generate ROS was shown as a contributing factor to the development of resistance using a xenograft mouse model by Bossis *et al.*^[120]^. According to their findings, ROS-dependent inhibition of the SUMO-conjugating enzymes was shown to induce apoptosis in chemo-sensitive AML cells, but in chemo-resistant cells, chemotherapeutics failed to activate the ROS/SUMO axis, and as a result, apoptosis through this pathway was not activated. In this study, diphenyleneiodonium (DPI), a NOX inhibitor (when used at low doses; otherwise, it has the propensity to inhibit other flavoproteins), prevented both DNR-induced loss of SUMO conjugates and apoptosis, and therefore, the activity of NOX was considered crucial for the success of chemotherapy. The following study demonstrated that suppression of oxidative stress by AMP-activated protein kinase (AMPK) protected murine AML cells, which provides further evidence supporting the correlation between low ROS and resistance^[121]^. Using a H_2_O_2_-sensitive marker and a murine animal model, Huang *et al.* demonstrated that the closer AML blasts are located to endosteal niche (and further from the vascular niche) and have low ROS, the AML cell population is enriched in CD34^+^CD38^-^ and enriched further following ARA-C treatment^[85]^. The gene expression pattern of these cells was associated with increased levels of enzymes leading to increased antioxidant molecules. As chemotherapy induces oxidative stress, it is logical to hypothesize that the AML cells with low ROS levels have an advantage for survival and show resistance to chemotherapy. Analysis of ARA-C-resistant cells from AML patient-derived xenografts (PDX) treated with ARA-C revealed high levels of ROS, increased mitochondrial mass, and a high OXPHOS status. The high ROS in these resistant cells was either a surrogate marker of resistance and elevated as a consequence of increased OXPHOS or had a direct role in resistance to ARA-C. The latter would be in contrast with the model from the aforementioned studies, where insufficient ROS was associated with resistance. In this AML PDX model of resistance, elevated OXPHOS and mitochondrial activity and increased reliance on fatty acid metabolism were demonstrated as the mechanism of resistance. Using a limited number of AML cell line models, Robinson *et al.* also demonstrated the significant ROS-specific metabolic alterations in sphingolipid metabolism, fatty acid oxidation, purine metabolism, amino acid homeostasis, and glycolysis which supported the role of ROS in directing metabolic changes in AML^[122]^. A group of AML patients with ARA-C resistance had high mitochondrial OXPHOS and were highly sensitive to VEN and ARA-C^[123-125]^. Following VEN and ARA-C treatment, a subpopulation of these cells developed an adaptive resistance associated with new alterations in OXPHOS mediated by the activation of alternative regulators of OXPH, such as TP53 and MITF^[126]^. The alteration of ROS following the new adaptation of OXPHO and its direct contribution to resistance was not investigated; however, targeting mitochondria through the inhibition of mitochondrial fusion, which damaged ROS production, led to cell cycle arrest^[127]^. As the inhibition of ROS is not separated from OXPHOS damage in mitochondrial fusion targeting, determining a specific role for ROS as a contributor to adaptive resistance is a task for future investigation. Based on these studies, it can be hypothesized that cells recruit protective measures to reduce the high production of ROS as they increase their OXPHO as a mechanism of resistance.

It can be argued that the inability of a subclone of AML cells to upregulate the generation of ROS results in resistance to chemotherapy. Alternatively, while the main mechanism of resistance is due to the high activity of mitochondria and altered metabolic pathways such as a shift to fatty acid metabolism, the emerging resistant clone produces high levels of ROS, but the contribution of high ROS levels is not well-characterized^[128]^. This was also illustrated in a xenograft mice model by Ma *et al.*, where chemotherapy resistance was associated with increased dependence of metabolism on OXPHOS in mitochondria and the inhibition of drug-induced ROS. According to this study, the metabolic reprograming by SIRT3 increased the reduced glutathione/oxidized glutathione ratio which reflects the enhanced antioxidant capacity^[129]^. In addition to controlling ROS levels, SIRT3 promotes fatty acid oxidation in LSC, and therefore, its inhibition leads to fatty acid accumulation in LSC, which could be cytotoxic. However, LSC can still protect themselves by upregulating cholesterol metabolism, and combined suppression of SIRT3 and cholesterol metabolism are required to overcome the SIRT3-dependent resistance to chemotherapy^[130]^. By increasing fatty acid metabolism, SIRT3 has been associated with resistance to VEN^[125]^ and therefore targeting SIRT3 could be considered one of the targets in VEN-resistant LSC. SIRT1, a cellular stress sensor, a deacetylase, and a negative regulator of TP53, has been shown to contribute to chemotherapy resistance in AML cells by reducing the intracellular level of O_2_^•-^^[131,132]^. FOXP1, a member of the FOXO transcription family, selectively enhances the expression of SIRT1 in committed but not HSC-enriched myeloid progenitors. FOXP1/SIRT1 pathway promotes the survival of AML cells, and in FLT3-ITD AML patients, SIRT1 expression level has been correlated with poor prognosis^[133]^. Another member of the SIRT family, SIRT2, increases G6PD activity by deacetylating its Lys403 residue which subsequently leads to the production of NAPDH through the PPP pathway. NADPH contributes to fatty acid synthesis and antioxidant process^[134,135]^. The high level of SIRT2 in clinical AML samples has been correlated with poor overall survival^[136]^. A potential role suggested for SIRT5, another member of the SIRT family, is by regulation of OXPHOS through enhancement of glutamine metabolism and as a potential therapeutic target in the AML cells which depend on SIRT5 for glutamine metabolism^[137]^.

### Source of ROS generation

The source of ROS generation in AML cells may influence the mechanism through which ROS contributes to pathogenesis and response to therapy. The role of ROS in the context of production source was described recently by Paolillo *et al.*, who reported a large increase in ROS in chemo-resistant AML cells and demonstrated the strong correlation with the increased level of the NOX2 subunits such as *CYBA*, *NCF1*, *NCF2*, *NCF4*, and *RAC2* along with elevated expression of NOX2 (*CYBB*) on the cell surface of these cells^[138]^. Using an AML cell line model (HL60), this group showed that upon development of resistance to DNR, *NOX2* expression increased, and the resistance could be overcome by inhibiting NOX2 either chemically or through gene targeting^[138]^.

Higher activity of NOX2 requires increased activity of other genes which provide co-factors or substrates for NOX2 activity. One of these factors is Nicotinamide phosphoribosyl transferase (NAMPT), which encodes an essential enzyme in the recycling of NAD, a critical co-factor for NADPH oxidase activity. In the analysis of the LSC in VEN/AZA-resistant patients compared to LSC from untreated AML patients, an increase in the activity of NAMPT was observed, which enabled the cells to use fatty acids to enhance oxidative phosphorylation to overcome sensitivity to VEN/AZA^[139]^. In an AML murine model, the generated superoxide by NOX2 stimulated BM stromal cells to transfer their mitochondria to AML blast cells through AML-derived tunneling nanotubes, resulting in increased AML cells survival^[140]^, indicating the generated ROS from non-mitochondrial source can boost OXPHOS by increasing the mitochondrial content of the AML cells.

### Interaction with microenvironment

The suppressive effect of the generated ROS on the immune system^[141]^ might be one of the mechanisms of enhanced resistance to therapy, although the damaging effect of ROS on DNA and aberrations of the DNA repair mechanisms have also been suggested as a potential mechanism of resistance to therapy^[142,143]^. One of the potential mechanisms of developing resistance by AML cells is to alter the metabolism and reliance of the pathways that are not targeted by the inhibitor. A recent *in vitro* study demonstrated that AML cells can acquire their substrate for metabolism from the mesenchymal stromal cells by the mediation of ROS. The generated ROS from AML cells were shown to enter the stromal cells through gap junctions and increase the glycolysis in the stromal cells, resulting in increased production of acetate and its release to their microenvironment. The released acetate was used by AML for the tricarboxylic acid cycle (TCA) and lipid biosynthesis^[144]^. This mechanism helps the AML cells to enhance the resources when extra energy is required.

In parallel to understanding the role of ROS in cancer development, relapse, and drug resistance, efforts are in progress to develop drugs that can target cancer cells by either inhibiting ROS in cancer cells when they depend on ROS for proliferation and viability or increasing ROS to toxic levels in cancer cells with poor buffers against oxidative damage. Table 3 summarizes some strategies for manipulating the redox (reduction/oxidation) balance to selectively target cancer cells.

**Table 3:** Clinically approved and under-investigation agents targeting oxidative stress

**Inhibitor**	**Mechanism of action**	**Drug**
NOX inhibitors	Target NOX enzymes involved in ROS generation	GKT137831^[145]^, GKT136901^[146]^, apocynin^[147]^
Mitochondrial antioxidants	Mitochondrial antioxidants are a class of compounds that selectively accumulate within the mitochondria to exert antioxidant effects	MitoQ^[148]^, MitoVitE^[149]^, MitoTempol^[150]^
SOD mimetics	SOD mimetics are synthetic compounds that mimic the activity of the endogenous antioxidant enzyme SOD	GC4419^[151]^, MnIIITE-2-PyP5+^[152]^
Peroxiredoxin inhibitors	The inhibitors of antioxidant peroxiredoxin enzymes lower the cell’s ability to neutralize endogenous hydrogen peroxide and lead to accumulation of ROS and oxidative stress which can trigger cancer cell apoptosis and enhance cytotoxic oxidative damage	Conoidin A^[153]^, Adenanthin^[154]^
TrxR inhibitors	TrxR regulates the antioxidant thioredoxin system that controls intracellular ROS levels. Inhibition of TrxR leads to oxidative stress due to attenuation of this cytoprotective system. This mechanism of induction of redox imbalance makes auranofin and other potential TrxR inhibitors attractive as anti-cancer drugs	Auranofin^[155]^
Lipid antioxidants	These antioxidants scavenge ROS and reactive nitrogen species	Polydatin^[156]^, quercetin^[157]^, ferulic acid^[158]^
PARP inhibitors	PARP enzymes detect DNA damage and synthesize PAR polymers to recruit repair factors. Oxidative stress results in single-strand DNA breaks activating excessive PARP. PARP inhibitors prevent this process, leading to an accumulation of DNA lesions. This excessive genomic instability triggers replication fork collapse and eventual apoptosis and cell death. The cancer cells with HR defects are selectively killed by PARP inhibition as they rely heavily on remaining backup repair mechanisms that are blocked	Olaparib^[159]^, veliparib^[160]^
NF-κB inhibitors	H_2_O_2_ has been shown to induce NF-κB through IKK activation, but the underlying molecular mechanisms for this activation have been proposed to be highly cell-type specific and involve different mechanisms^[161,162]^. Strategies directed against individual targets in the NF-κB signaling pathway, including antioxidants and pharmacological inhibitors of IKK or the 26S proteasome complex, have shown considerable efficacy in improving and recovering tissue injury in animal models^[163]^	Thiol antioxidants (N-acetylcysteine, lipoic acid)^[164]^, curcumin^[165]^
Nrf2 activators	Nrf2 activators are indirect antioxidants, as by activating Nrf2 signaling, they can stimulate the intrinsic cellular antioxidant defenses through ARE-driven gene expression	Sulforaphane^[166]^
ROS-activated prodrugs	ROS-activated prodrugs are activated by ROS inside cancer cells to release cytotoxic drugs. The prodrug itself is non-toxic and contains a redox-sensitive linker region that can be cleaved by ROS. As cancer cells intrinsically generate higher oxidative stress and ROS, prodrugs are cleaved and activated within cancer cells and release the active cytotoxic drug. Meanwhile, in normal cells with lower ROS, the prodrug remains inactivated and therefore causes little toxicity	Boron-based ROS-activated prodrugs, ROS-activated nitrogen mustard prodrugs, ROS-activated quinone methide prodrugs^[167]^

NADPH: Nicotinamide adenine dinucleotide phosphate; NOX: NADPH oxidase; ROS: reactive oxygen species; SOD: superoxide dismutase; TrxR: thioredoxin reductase; PARP: poly (ADP-ribose) polymerase; IKK: IκB kinase; ARE: Antioxidant Response Element.

## CONCLUSION

The role of ROS in the pathogenesis of AML, as well as their influence on responses to chemotherapy and the likelihood of relapse, remains to be better understood. This knowledge gap can be attributed to the diverse roles that ROS play in cell metabolism and signaling pathways. Current data suggests that both high and low levels of ROS can contribute to therapy resistance and the potential for relapse. Furthermore, the sources of ROS production may affect AML cell survival following chemotherapy. Lower levels of ROS are associated with the maintenance of leukemia stemness, reduced sensitivity to chemotherapy, and a higher risk of future relapse. In contrast, NOX2-mediated ROS generation may enhance AML cell survival by providing an additional source of energy. This occurs through mediating the transfer of mitochondria and essential metabolites from stromal cells in the microenvironment to AML cells. The ROS generated by NOX2 may also induce metabolic alterations in AML cells, favoring alternative pathways to protect against the harsh environment created by chemotherapy. Moreover, ROS generated by AML cells can alter the microenvironment and suppress the immune components within it, further promoting AML cell survival. The complexity and diversity of ROS generated by AML cells make directly targeting ROS a challenging task for therapeutic purposes. Recent increasing evidence highlights the significance of NOX2 in therapy resistance. Preliminary data even suggest a predictive role for NOX2 expression in resistance to therapy. The availability of specific NOX2 inhibitors makes it an attractive target for further *in vitro* and *in vivo* investigations as a potential therapeutic option. Alternatively, conducting a detailed analysis of the signaling pathways altered by ROS and exploring them as potential therapeutic targets, rather than directly targeting ROS or their substrates, offers alternative avenues for research in the development of additional therapeutic targets in AML.

## DECLARATIONS

### Copyright

© The Author(s) 2024.
